# MendelVar: gene prioritization at GWAS loci using phenotypic enrichment of Mendelian disease genes

**DOI:** 10.1093/bioinformatics/btaa1096

**Published:** 2021-01-16

**Authors:** M K Sobczyk, T R Gaunt, L Paternoster

**Affiliations:** MRC Integrative Epidemiology Unit, Bristol Medical School, University of Bristol, Bristol BS8 2BN, UK; MRC Integrative Epidemiology Unit, Bristol Medical School, University of Bristol, Bristol BS8 2BN, UK; MRC Integrative Epidemiology Unit, Bristol Medical School, University of Bristol, Bristol BS8 2BN, UK

## Abstract

**Motivation:**

Gene prioritization at human GWAS loci is challenging due to linkage-disequilibrium and long-range gene regulatory mechanisms. However, identifying the causal gene is crucial to enable identification of potential drug targets and better understanding of molecular mechanisms. Mapping GWAS traits to known phenotypically relevant Mendelian disease genes near a locus is a promising approach to gene prioritization.

**Results:**

We present MendelVar, a comprehensive tool that integrates knowledge from four databases on Mendelian disease genes with enrichment testing for a range of associated functional annotations such as Human Phenotype Ontology, Disease Ontology and variants from ClinVar. This open web-based platform enables users to strengthen the case for causal importance of phenotypically matched candidate genes at GWAS loci. We demonstrate the use of MendelVar in post-GWAS gene annotation for type 1 diabetes, type 2 diabetes, blood lipids and atopic dermatitis.

**Availability and implementation:**

MendelVar is freely available at https://mendelvar.mrcieu.ac.uk

**Supplementary information:**

Supplementary data are available at *Bioinformatics* online.

## 1 Introduction

The last decade has delivered a bounty of genetic data due to advances in high-throughput DNA sequencing and genotyping. On the one hand, this has led to dramatic advances in investigation of the genetic basis of complex, polygenic disease and traits with 9407 studies featured in the GWAS Catalog as of October 2020 (Buniello *et al.*, 2019). However, the detection of a GWAS signal alone does not identify the causal gene at a locus and so substantial bioinformatics and experimental effort is still required to convert such findings into useful biological insight. At the other end of the spectrum, Mendelian monogenic disease research has benefitted tremendously from recent sequencing methods, which helped to detect the causal genes in >1000 Mendelian conditions (Bamshad *et al.*, 2019). In contrast to complex trait loci, large-effect sizes and typically missense consequences of Mendelian perturbations mean the causal gene is more easily detected using statistical methods alone, resulting in a direct link between phenotype and gene. In MendelVar, we utilize these direct links between phenotypes and genes from Mendelian traits to aid in identifying causal genes and pathways implicated in GWAS of complex traits.

MendelVar is a unique comprehensive tool linking the information obtained about gene function in Mendelian disease research to help inform candidate gene prioritization in GWAS. First, it makes it easy to find associations of a given set of genomic intervals or single nucleotide polymorphisms (SNPs) with Mendelian disease in a range of databases. Second, it tests for enrichment of disease phenotype and gene ontology terms associated with detected Mendelian disease. MendelVar is presented as a freely available and regularly updated webserver accessible on: https://mendelvar.mrcieu.ac.uk/.

The number of genes with a known disruption resulting in a Mendelian disorder currently totals 4229 genes in Mendelian Inheritance In Man (OMIM) (October 2020). With ∼300 new Mendelian phenotypes added to the database every year, there is a large scope for an increase in numbers for genes associated with Mendelian disease. Therefore, MendelVar will be regularly updated with new findings on Mendelian disease genes.

Many common complex diseases (caused by hundreds of loci with small effect) contain a small subcohort of individuals with monogenic large-effect disruption in key genes driving complex disease—with examples including coronary artery disease, diabesity, obesity and autism (Chong *et al.*, 2015). Since small-effect mutations in the affected genes circulate in the general at-risk population, function of genes affected in the monogenic forms can inform us about the main biological processes involved in complex disease aetiology and aid therapeutic development.

Freund *et al.* (2018) have shown that gene sets with confirmed phenotypically matching or related Mendelian lesions are ∼27 times more likely to be enriched among all GWAS genes across 62 human traits compared to phenotypically unrelated sets of Mendelian disease genes. Examples include enrichment of growth defect genes in the height GWAS or immune dysregulation genes in a range of inflammatory conditions, such as inflammatory bowel disease. In general, Mendelian disease genes show enrichment among genes flanking the low *P*-value disease GWAS loci and their occurrence positively correlates with association strength (Chong *et al.*, 2015). Widespread comorbidity has also been detected between Mendelian disease and complex disease (Blair *et al.*, 2013) including cancer (Melamed et al., 2015), which can potentially be driven by pleiotropy (Pividori et al., 2019). The established involvement of certain Mendelian disease genes in complex traits has started to become utilized in evaluating GWAS gene prioritization algorithms (Barbeira *et al.*, 2019; Guo *et al.*, 2019) and indeed, in gene prioritization itself (Schlosser et al., 2020). MendelVar aims to simplify this process of integrating information about Mendelian disease to prioritize candidate causal genes at GWAS loci. It has been indicated that Mendelian disease-linked genes make for more successful drug targets (King *et al.*, 2019; Nelson et al., 2015) and so integration of Mendelian disease data may be especially useful in prioritizing the loci with Mendelian disease evidence for pharmaceutical interventions.

MendelVar allows quick assessment of the likely impact of Mendelian disease-related genes from specified genomic regions (identified by GWAS or other means) on the user's complex phenotype of interest. It lists the details of all the Mendelian disease genes found in the input genomic intervals, extracted from OMIM and similar databases, as well as the closest rare mutations mapped to them available in ClinVar. INRICH is then used for calculating the enrichment of Disease Ontology (DO) and Human Phenotype Ontology (HPO) terms amongst the background of all Mendelian disease-related genes, giving the researcher an overview of any possible shared phenotypic features of identified Mendelian genes with the trait of interest, e.g. in terms of anatomy.

In this paper, we first describe the process of identification, filtering and integration of Mendelian disease data sources. We compare MendelVar with similar tools in terms of analytical features and the breadth of data mined. Following that, we present different possible MendelVar workflows and apply these to three example traits.

## 2 Materials and methods

Full details of how the MendelVar database was constructed is available in the Supplementary Material, but briefly:

Disease-gene relationships from OMIM, Deciphering Developmental Disorders Study (DECIPHER), Orphanet and Genomics England were extracted and integrated. Gene coordinates were defined according to the canonical transcript reported in APPRIS or GENCODE. In addition, variant-disease relationships were also extracted from ClinVar to enable identification of variants of interest in the regions under investigation. The disease genes were mapped to a range of ontologies (described in the implementation) to enable relevant phenotype searching within the results, and also for testing of enrichment of ontology terms across multiple genomic regions with INRICH.

## 3 Results

### 3.1 Implementation

#### 3.1.1 Integration of MendelVar data sources

The paramount reference for description of Mendelian disease and their causal genes is the (Online) OMIM database (Amberger *et al.*, 2019). MendelVar uses all the confirmed gene-disease relationships featured in OMIM and complements it with three more specialist data sources for Mendelian disease: Orphanet (Rath et al., 2012) (a database centred on rare, typically monogenic disease), expertly curated gene panels used for diagnostics from Genomics England PanelApp (Martin et al., 2019) and results from the on-going Deciphering Developmental Disorders Study (DECIPHER)—whose aim is to identify *de novo* microgenomic rearrangements responsible for undiagnosed developmental delay disorders (Firth *et al.*, 2009).

While OMIM is the established reference for mapping Mendelian disease to genes, adding gene-disease associations from DECIPHER, Orphanet and Genomics England panels allows discovery of more genes compared to OMIM alone (Table 1). The 12 344 gene-disease relationships in MendelVar contain 4843 unique genes, 552 of them not assigned to any Mendelian disease in OMIM. Integration of the four data sources resulted in 12 344 gene-disease relationships, compared to 6471 present in OMIM alone. Although there is some redundancy, across Orphanet, DECIPHER and Genomics England, we found at least 1925 gene-disease associations (either at an individual disease level or same phenotypic series level) that were not in OMIM (i.e. counting only entries that have both gene and disease OMIM ID assigned).

**Table 1.** Comparison of MendelVar with its Mendelian disease source databases

	OMIM	Orpha^a^	GE^b^	DECIPHER	MendelVar
Number of genes	4229	4202	3244	2198	4843
Number of diseases	5618	3817	4430	2873	7532
Number of gene- disease associations	6471	7619	5019	3679	12 344

a Orphanet.

b Genomics England.

MendelVar includes short disease descriptions sourced from OMIM, Orphanet, Uniprot (Breuza *et al.*, 2016) and DO (Schriml et al., 2019). Out of 12 344 gene-disease entries in the MendelVar database, 9899 contain a disease description, amounting to 5329 unique disease descriptions.

In addition, MendelVar cross-references input genomic intervals against ClinVar (Landrum et al., 2019) for pathogenic or likely pathogenic variants implicated in Mendelian disease allowing identification of mostly genic variants and repeats in linkage-disequilibrium (LD) with region of interest. Due to small differences in genome builds, 134 347 and 130 123 ClinVar variants are present in the GRCh37 and GRCh38, respectively.

Following extraction of Mendelian disease-linked target genes from the MendelVar database, MendelVar provides enrichment testing of any specific phenotypic class associated with the disease in question. This is done by first annotating the target genes with all the ontology terms associated with all the Mendelian diseases caused by any mutation in the gene. MendelVar provides easy enrichment testing using human DO and HPO, their respective slim ontologies and Freund *et al.* (2018)’s 20 Mendelian gene sets based around broad phenotypic categories. The DO focuses on defining a hierarchy of disease aetiology classes which include affected anatomical entity and part of metabolism, amongst others. The main remit of HPO (Köhler et al., 2019) is the classification of clinical symptoms and abnormalities associated with disease.

In MendelVar, HPO terms were collected first from OMIM, and then complemented with Orphanet, Decipher DDG2P and official HPO annotations, which altogether contributed HPO terms to 423 diseases with no previous HPO terms in OMIM. Similarly, DO terms were first mined from OMIM, followed by Orphanet and the official DO annotation. The latter two sources provided DO annotation to 579 diseases with no previous annotation in OMIM. Out of 12 344 gene-disease entries in the MendelVar database, 10 215 (6284 unique diseases) have at least one HPO term assigned and 6574 (3564 unique diseases) have a DO term assigned. In total, we include 9209 distinct HPO and 4651 DO terms in the MendelVar database. The mean number of all HPO terms and DO terms is 149.2 (20.3 independent leaf terms) and 10.5 (2 independent leaf terms) per ontology-annotated gene, accordingly.

MendelVar also incorporates the Gene Ontology (Carbon *et al.*, 2019), Pathway Commons (Cerami *et al.*, 2011), Reactome (Fabregat *et al.*, 2018) and ConsensusPathDb (Herwig *et al.*, 2016) allowing custom testing for enrichment of molecular and biochemical gene pathways against the null background of all Mendelian disease genes in the human genome.

#### 3.1.2 User input for MendelVar webserver

The MendelVar webserver accepts a maximum input of 10 000 genomic positions or intervals, with a maximum interval size of 20 Mbp.

**Fig. 1.**
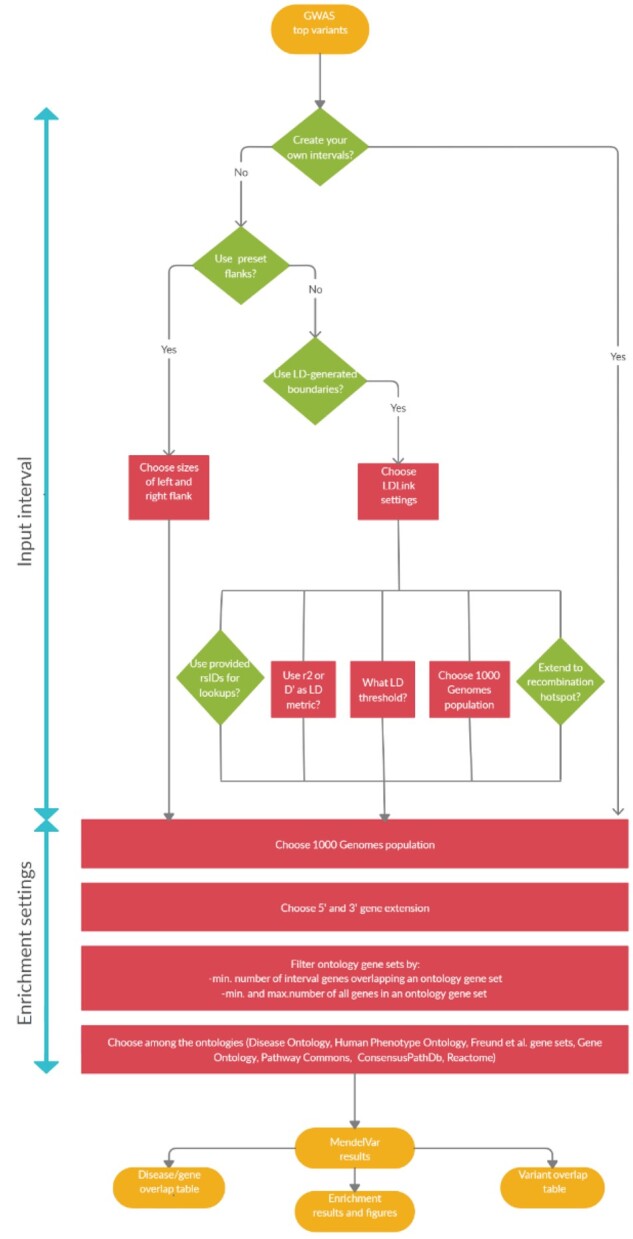
A flowchart demonstrating three possible user routes through MendelVar: (**a**) *left*: MendelVar generates fixed genomic intervals using preset left and right flanks against a user-submitted list of genomic positions; (**b**) *centre*: MendelVar generates flexible genomic intervals using LD pattern in the region around each user-submitted position/variant rsID; (**c**) *right*: MendelVar accepts user-submitted genomic intervals. The genomic intervals generated or obtained from user are subsequently bisected with coordinates for genes and variants known to cause Mendelian disease. Ontology terms associated with Mendelian disease in HPO, DO are propagated to causal genes and are tested for enrichment among target genes in input genomic intervals. MendelVar also provides an option for enrichment testing with Gene Ontology and biological pathway databases

MendelVar offers three general routes through the pipeline depending on the type of user input. The user can either submit predefined GRCh37 or GRCh38 genomic intervals based on their own analysis pipeline (Fig. 1  *right*) or a list of single genomic positions (e.g. GWAS lead SNPs) from which MendelVar will create the intervals to be used. These intervals can be created in two ways. Either, user-specified left and right flanks can be generated around the input positions, up to a maximum of 10 Mbp in each direction (Fig. 1  *left*), or, flexible LD-based intervals can be created using the LDlink LDproxy app (Machiela and Chanock, 2015), either through dbSNP rsIDs or single positional coordinates (Fig. 1  *centre*) from GRCh37 (hg19) or GRCh38 (hg38) assemblies. Using an LD statistic of choice: *r*^2^ or *D*ʹ, LD is calculated within the 1 Mbp window centred on the input SNP with LDlink. Boundaries around the input variant are generated by finding the most distant upstream and downstream variant meeting the specified minimum LD threshold (between 0 and 1) in the selected target 1000 Genomes population: CEU (Utah residents of northern European descent), EUR (European), EAS (East Asian), SAS (South Asian), AFR (African) or AMR (Ad-mixed American). This generated interval can be extended to the nearest recombination hotspot with a recombination rate >3 cM/Mb (Myers et al., 2008) based on HapMap II. As LDlink accepts only GRCh37 coordinates, we initially map user-submitted GRCh38 coordinates to GRCh37 coordinates with the UCSC liftOver tool (Haeussler *et al.*, 2019). Therefore, the last step in the case of user input in GRCh38 coordinates is to convert the LDlink-create*d*.

GRCh37 intervals, optionally extended to the nearest recombination hotspot, back to GRCh38 coordinates using the UCSC liftOver tool.

#### 3.1.3 Identification of overlapping mendelian genes

Genes are defined as overlapping with the genomic intervals based on coordinates of canonical APPRIS (Rodriguez et al., 2013) transcript isoforms if available or alternatively the longest transcript for a given gene; the overlap step is performed with GIGGLE (Layer et al., 2018).

Users can extend the gene region by up to 20 kbp in upstream or downstream direction since most of the strong eQTLs (expression quantitative trait loci) regulating gene expression are found in that region (Veyrieras et al., 2008). It is not recommended to extend the gene region too much, as this can result in more genes overlapping each other and being collapsed by INRICH (Lee et al., 2012), which will then result in a loss of power in the *‘*gene*’* INRICH mode (*see below*).

#### 3.1.4 Enrichment testing

Following identification of Mendelian disease-associated genes overlapping the input genomic intervals, MendelVar allows testing for enrichment of terms associated with those genes relative to the background of all Mendelian disease-associated genes in the genome. Of particular interest in GWAS for complex traits (and uniquely compared to other tools), we allow simultaneous testing using HPO, DO and Freund *et al.* (2018) gene sets. To give the user an overview of general categories associated with both ontologies and boost statistical power, MendelVar includes the official DO slim (24 terms) and custom-generated HPO slim (25 direct descendants of the root term *‘*Phenotypic abnormality*’*). In addition, general gene enrichment testing is available with Gene Ontology and its slim version, and pathway enrichment testing with ConsensusPathDB, PathwayCommons and Reactome.

Enrichment testing in INRICH can be run in two modes: *‘*gene*’* and *‘*interval*’*. In the *‘*gene*’* mode, the enrichment statistic is calculated over the number of genes in all intervals overlapping a given ontology term. In the *‘*interval*’* mode, the enrichment statistic is calculated over the number of intervals with at least one gene (regardless of gene number within interval) overlapping a given ontology term. In general, *‘*gene*’* mode should result in higher power to detect enrichment in the context of typical MendelVar usage, if we expect multiple genes with related functions to cluste*r*.

#### 3.1.5 Mendelvar output

**Fig. 2.**
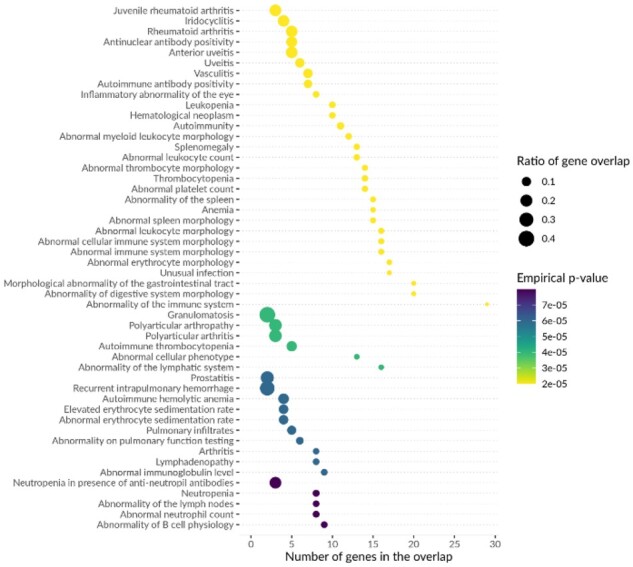
Top enrichment results for HPO terms among Mendelian disease genes located within LD-based intervals around lead SNPs in Onengut-Gumuscu *et al.* (2015) T1D GWAS. An example of a summary figure produced by MendelVar pipeline depicts top 50 most significant terms in the ontology (*y* axis), sorted first by empirical *P*-value followed by the number of genes overlapping the ontology term in the tested genomic intervals in case of ties (also shown independently on the *x* axis). The size of each data point represents ratio of gene overlap, i.e. number of genes found in the tested genomic intervals divided by all Mendelian genes annotated with that ontology term available in the genome. Each data point is coloured according to empirical *P*-value for enrichment significance

**Fig. 3.**
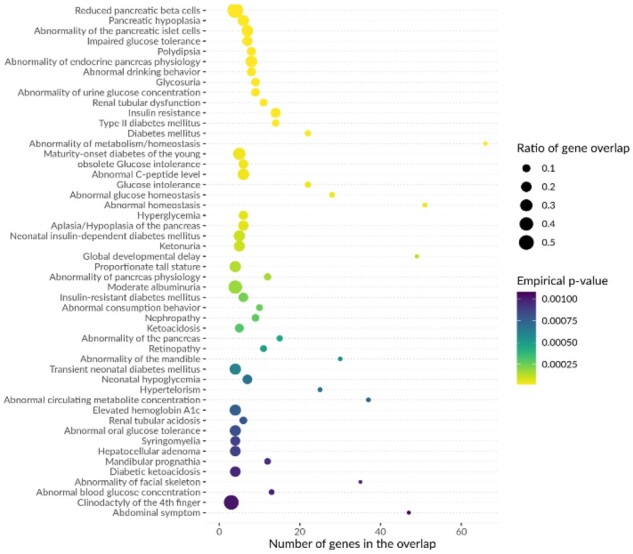
Top enrichment results for HPO terms among Mendelian disease genes located LD-based intervals around lead SNPs in Xue *et al.* (2018) T2D GWAS. in a single locus. However, one consequence in the context of GWAS is that this enrichment value will be biased by the same GWAS signal counted multiple times

The average run time using 100 single variant positions, interval generation with LDlink and enrichment testing using all ontologies is 5 h due to resampling and bootstrapping steps in INRICH. Two main results tables are produced, which detail the overlap of input genomic intervals with Mendelian-disease causing genes and variants, respectively (see example Supplementary Tables S1 and S2 and abbreviated in Table 2, full results for example traits in Datasets S1–S7). Results of enrichment testing using each ontology are provided in individual tables and are summarized in a set of figures (see example Figs 2 and 3, full results for example traits in Datasets S1–S7).

**Table 2.** Output from the MendelVar main results table (select columns out of 20)

ID	Interval tested	Gene name	Disease name	Link
rs2296173 … rs1899951 rs3802177 rs753270	1:39085570–39624273 3:12245220–12433589 8:117171419-117206282 10:79200553-79235799	*MACF1* *PPARG* *SLC30A8* *ZMIZ1*	Lissencephaly 9 with complex brainstem malformation; defects in neuronal migration and axon guidance; posterior-predominant lissencephaly broad flat pons and medulla-midline crossing defects syndrome Insulin resistance, susceptibility to; leanness, inherited; lipodystrophy, familial partial, type 3; carotid intimal medial thickness 1; thyroid carcinoma, Hurthle cell; Giant cell glioblastoma; Gliosarcoma Insulin resistance, susceptibility to neurodevelopmental disorder with dysmorphic facies and distal skeletal anomalies; neurodevelopmental disorder with dysmorphic facies and distal limb anomalies; syndromic neurodevelopmental disorder	https://www.omim.org/entry/618325 https://www.omim.org/entry/125853; https://www.omim.org/entry/601665; https://www.omim.org/entry/604367; https://www.omim.org/entry/609338 https://www.omim.org/entry/125853 https://www.omim.org/entry/618659

*Note*: An abbreviated version of the full disease-based results table (Dataset S2) from the T2D MendelVar analysis.*

We include the first locus in the genome (as an unselected example) and then the three loci for which Grotz *et al.* (2017) have reported causal genes. Sometimes the ontological connection with the trait of interest is apparent, whilst in other cases the genes returned may be false positives. The enrichment results can help to distinguish these.

### 3.2 Comparison with related tools

We compared MendelVar to seven most closely matched applications, which also facilitate the use of Mendelian disease data in human genetics research (Supplementary Table S3). These tools show differences in accepted inputs, datasets included and overall philosophy and goals of the analysis. Broadly scoped tools such as FUMA (Watanabe et al., 2017) provide an excellent starting point for GWAS annotation by providing access to rich compendium of resources and analyses, such as gene expression, chromatin interactions and eQTLs. However, by the virtue of being so wide-ranging, they provide only a limited overview of the GWAS link to Mendelian disease and their phenotypes. Packages such as VarfromPDB (Cao *et al.*, 2017), while integrating a selection of the datasets present in MendelVar, are built on a reverse premise and mine for variants and genes associated with a phenotype/disease input by the user. Tools such as MARRVEL (Wang et al., 2017), FUMA (Watanabe *et al.*, 2017) and DisGeNET (Piñero et al., 2017) annotate input genes with matching Mendelian disorders from only a subset of databases integrated in MendelVar. Whilst the Open Targets Platform (Carvalho-Silva *et al.*, 2019) does collate Mendelian gene evidence for linking genes to disease, this is not a feature of its variant-based search. None of these four tools includes annotation with the full set of human DO and Phenotype Ontology terms or features enrichment testing of these terms.

Another comparison group consists of tools such as clusterProfiler (Yu et al., 2012), DAVID (Huang *et al.*, 2009) and Enrichr (Kuleshov et al., 2016). These are popular gene set enrichment packages widely employed in interpreting long gene lists resulting from high-throughput genomics and transcriptomics experiments. Despite a large number of compatible ontologies, they never feature more than one of the key ontologies (DO, HPO) available in MendelVar and do not carry out the background gene selection sampling taking into account genomic confounders such as SNP density and gene size/density which are required for appropriate GWAS analysis. Simulations have shown that ignoring these confounders in enrichment analysis in GWAS studies and assuming independence can result in up to 100% type I error in some cases, whilst it never exceeds the nominal 5% in INRICH (Lee *et al.*, 2012). Finally, in terms of flexibility of user input, other than Enrichr, MendelVar is the only enrichment tool to accept genomic coordinates.

We expect that MendelVar users may also utilize other tools in order to triangulate evidence across different data sources, but MendelVar provides the most comprehensive analysis of the relevant Mendelian disease gene data and aims to do so in a flexible and user-friendly way.

### 3.3 MendelVar use cases

#### 3.3.1 Type 1 and type 2 diabetes GWAS

In order to evaluate MendelVar’s usefulness in polygenic traits with varying genetic architecture, we applied it to analysis of type 1 diabetes (T1D) and type 2 diabetes (T2D) GWAS. These two common diseases can be contrasted as, despite a similar clinical endpoint (hyperglycaemia), they differ in general disease mechanism and genetic architecture (Timpson et al., 2018). Susceptibility to T1D has been shown to depend on lower-frequency common variants, many with large-effect size, whereas high-frequency common variants of small-effect size contribute to the overall risk of T2D.

MendelVar was used to generate LD-based intervals (*r*^2^>0.8 in Europeans) around lead SNPs downloaded from the GWAS Catalog (Milano et al., 2017). Genome-wide significantly enriched terms in DO and HPO matched known disease biology. In the case of T1D (Onengut-Gumuscu et al., 2015), we found enrichment of terms (Figs 2 and S1, Dataset S1): *abnormality of digestive system morphology*, *glucose metabolism disease*, terms related to T1D’s autoimmune root cause (*autoimmune disease*, *primary immunodeficiency disease*) and secondary symptoms of the disease such as vascular problems (*vasculitis*) (Schram et al., 2003), diabetic eye disease (*inflammatory abnormality of the eye*, *anterior uveitis*) (Watanabe et al., 2019). T2D (Xue et al., 2018) results showed enrichment of terms (Figs 3 and S2, Dataset S2) associated with insulin and glucose imbalance (*insulin resistance*, *glucose metabolism disease*, *impaired glucose tolerance*, *glycosuria*, *polydipsia*) and pancreas dysfunction (*abnormality of endocrine pancreas physiology*, *pancreatic hypoplasia*, *abnormality of the pancreatic islet cells*). In both T1D and T2D, we found enrichment of monogenic forms of diabetes, as expected (Yang and Chan, 2016)—2 genes in T1D (*RASGRP1*, *INS*) and 8 in T2D (*HNF1B*, *GCK*, *WFS1*, *KCNJ11*, *ABCC8*, *HNF1A*, *PPARG*, *SLC2A2*). We also found three Mendelian disease genes (Table 2) which were listed as ‘solved’ T2D loci with experimental evidence in a recent review (Grotz *et al.*, 2017). However, in one example, MendelVar highlights a known causal gene (ZMIZ1), but the Mendelian disease is not phenotypically aligned with the GWAS trait. Comparison of a number of Mendelian disease genes annotated with the enriched DO and HPO terms revealed *a* higher relative rate in T1D—32 genes assigned to 25 loci (out of 51 GWAS loci, 49%) versus 38 genes assigned to 29 loci (out of 150 GWAS loci, 19.3%) in T2D. The rate disparity decreased when considering any overlapping Mendelian disease genes: 47 genes were detected at 31 loci in T1D (60.8%) compared to 109 genes at 68 loci in T2D (45.3%).

#### 3.3.2 LDL And HDL plasma concentration GWAS

Since there are likely to be ancestral biases in Mendelian gene databases similar to those found in populations sampled in GWAS (Mills and Rahal, 2020), we wanted to investigate whether use of MendelVar on GWAS of the same traits in samples with different ancestries (but with comparable sample size) produced similar results in terms of genes identified and enrichment of annotated terms. In addition, low-density lipoprotein (LDL) and high-density lipoprotein (HDL) GWAS provide further examples of quantitative traits with a broad spectrum of effect sizes (Timpson *et al.*, 2018). Having downloaded the lead SNPs from the GWAS Catalog for each trait in European (LDL *n* = 85 491, HDL *n* = 89 614) (Teslovich et al., 2010) and East Asian (LDL *n* = 72 866, HDL *n* = 70 657) (Kanai *et al.*, 2018) GWAS, we followed the MendelVar route depicted in Figure 1b of generating LD-based intervals (*r*^2^>0.8) using a reference from European or East Asian 1000 Genomes population, respectively.

In general, we found genome-wide significant enrichment of HPO and DO terms to be shared across ancestries for LDL and HDL. A third of GWAS lipoprotein loci have been previously linked to genes directly involved in plasma lipoprotein metabolism, mutations in which frequently cause familial dyslipidemias (Dron and Hegele, 2016). Unsurprisingly, for LDL, we found enrichment of genes associated with terms such as: *familial hyperlipidaemias*, *increased/abnormal LDL-cholesterol concentration*, *xanthelasma* as well as terms related to known cardiovascular consequences of unbalanced LDL concentration (Wadhera et al., 2016): *arterial calcification*, *myocardial steatosis*, *cerebral artery atherosclerosis*, *coronary artery aneurysm* (Datasets S3 and S4). Similar terms but correctly identifying *decreased/abnormal HDL cholesterol concentration* were found to be enriched across HDL GWAS (Datasets S5 and S6).

As direct comparison between GWAS would be biased by the number of loci detected in each study and the clumping strategy used to define independent loci, we carried out repeated resampling of SNPs from independent loci to make the rate of MendelVar annotation more comparable (Table S4).

On average, 64.5% of loci in European LDL GWAS and 47% loci in East Asian GWAS were linked to any Mendelian disease. However, in HDL GWAS, the proportions were reversed across populations: 45.2% in European with any Mendelian disease annotation compared to 55.2% in East Asian. We found the same direction of difference when focussed only on diseases associated with HPO and DO terms enriched within either of the two populations.

#### 3.3.3 Atopic dermatitis GWAS

Lastly, we believe that MendelVar could potentially be useful in investigating subsignificant GWAS loci, to determine which loci (of all those that reach a suggestive *P*-value threshold) also have consistent independent evidence implicating loci gene with the disease. As an example, we investigated 157 loci with *P*-value of between >5×10^−8^ and <1×10^−4^ in the EAGLE atopic dermatitis (eczema) GWAS (Paternoster et al., 2015), and for each signal we defined a strict LD-based interval thresholded at *r*^2^>0.6 (Dataset S7). Among 72 Mendelian disease genes overlapping the intervals, 34 are associated with the general *abnormality of the skin* HPO term and 6 with the more specific top hit *abnormality of epidermal morphology*. On further investigation, one of these had evidence of eQTL colocalization (Giambartolomei *et al.*, 2014) in the skin: the 18q21.33 locus represented by the lead SNP rs35112771 (intronic) with a *P*-value of 2.1×10^−5^. The region showed convincing evidence of colocalization (83.5%) with *SERPINB7* eQTLs only in the sun-unexposed skin tissue type of GTEx study ver. 7 (Ardlie *et al.*, 2015). Crucially, *SERPINB7*’s expression is elevated in the epidermis, and nonsense mutations in the gene cause Nagashima-Type Palmoplantar Keratosis, characterized by well-demarcated diffuse hyperkeratosis with redness on palms and feet (Kubo et al., 2013). This phenotype is widely found in a range of eczematous conditions (Agner *et al.*, 2015; Lee et al., 2009; Thestrup-Pedersen et al., 2001) and would suggest that common mutations affecting *SERPINB7* expression could partially explain the missing heritability in atopic dermatitis GWAS.

## 4 Discussion

MendelVar is a unique webserver/pipeline dedicated to automatic assessment of the genetic and phenotypic link between Mendelian disease genes and hits at complex trait GWAS loci. It maps Mendelian disease to relevant variants and disease phenotypes as well as descriptions from a comprehensive sampling of public databases reporting Mendelian gene-disease associations. MendelVar’s rich database contains 4843 unique genes, 12 344 gene-disease relationships—80.2% with a disease description, 82.8% with HPO annotation, 53.2% with DO annotation; all complemented by ∼130 000 ClinVar rare variants. The Mendelian disease phenotypes and other gene ontology terms are then tested for enrichment in a GWAS-suitable framework. These features are integrated into a streamlined process beginning with input of genomic coordinates or variant rsIDs by the user. MendelVar delegates to the user a great deal of flexibility regarding workflow: allowing definition of a crude fixed interval around input genomic positions (GRCh38/hg38 or GRCh37/hg19), mapping positions or rsIDs to flexible LD-based regions or input of custom genomic intervals derived using independent methods. Our goal was to integrate all publicly available data sources mapping Mendelian disease to genes; hence, our inclusion of Orphanet, Genomics England PanelApp and DECIPHER to supplement the chief authority on Mendelian disease—OMIM. This strategy proved beneficial as MendelVar includes 629 novel genes and at least 1925 new gene-disease associations in addition to those provided by OMIM. For instance, *CREB3L3* and *CDX1* are not assigned any disorders on OMIM but are on the *‘*severe hypertriglyceridaemia*’* panel and *‘*nonsyndromic familial congenital anorectal malformations*’* panel, respectively, in the Genomics England PanelApp.

The absence of given gene-disease connections in OMIM may result from a delay due to its reliance on literature rather than clinical data, but also OMIM’s stricter requirements for inclusion and redundancy. Some links in the non-OMIM sources and supported by the older literature have been deemed not strong enough to deserve a separate entry in OMIM, e.g. the link between *KCNJ8* and Cantú syndrome (Cooper *et al.*, 2014) reported in DECIPHER or a link between *PLXND1* and Moebius syndrome in Orphanet (Tomas-Roca et al., 2015). Each gene-disease entry in the MendelVar results table lists all the data sources supporting the causal link, allowing case-by-case assessment by the user.

Some information is likely to be partially or wholly redundant due to different criteria for assignment to related conditions. For instance, DECIPHER reports *SATB2* to be linked to simply *‘*cleft palate*’*, whereas in OMIM the gene is mapped to Glass syndrome, for which one of the hallmarks is cleft palate. Similarly, Orphanet can present alternative assignment of genes to rare diseases relative to OMIM. For instance, *TRDN* is assigned to Romano–Ward syndrome on Orphanet and *‘*Ventricular tachycardia, catecholaminergic polymorphic, 5, with or without muscle weakness*’* on OMIM, despite the fact that Romano–Ward syndrome is also present in the OMIM database; both diseases are forms of cardiac arrhythmias.

Care needs to be taken when interpreting ontology enrichment results and enriched terms need to be cross-referenced with target trait phenotypes due to false positives stemming from horizontal pleiotropy. For instance, extensive overlap in the genetic aetiology of T1D with rheumatoid arthritis (RA) has been documented (Onengut-Gumuscu *et al.*, 2015). In line with this, our MendelVar analysis of the T1D GWAS finds significant enrichment of DO and HPO terms associated with RA such as: *rheumatoid arthritis*, *autoimmune disease of musculoskeletal system*. Similarly, while abnormality in the level of circulating neutrophils and platelets has been detected in T1D (Valle et al., 2013), more specific blood cell type abnormalities were, likely incorrectly, enriched in the MendelVar analysis. In this scenario, while the enriched terms may be false, the underlying shared genetic component could informatively point to a shared causal gene, such as *PTPN22* in T1D and RA (Bottini *et al.*, 2006).

Integration of Mendelian disease genetics into prioritization of genes at GWAS loci may have a positive impact on drug discovery pipelines. Studies have shown that the probability of progression along a drug development pipeline is greater for target-indication pairs with genetic evidence, and that OMIM genetic evidence has a greater effect on approval than GWAS evidence, e.g. phase I to approval progression risk ratio for GWAS = 1.4 (95% CI 1.1–1.7), OMIM = 2.7 (95% CI 2.4–3.1) (King *et al.*, 2019; Nelson *et al.*, 2015). Thorough analysis has demonstrated that this difference is unlikely to be due to a statistical artefact or reverse causation, whereby the OMIM entry is influenced by the knowledge of successful drug trials (King *et al.*, 2019). However, the higher probability of progression along drug development pipelines for OMIM genes may be influenced by Mendelian conditions having larger genetic risk factor effects than common GWAS traits (rather than just increased likelihood of identifying the true causal gene) and so it remains to be seen whether the use of Mendelian genetic data to link GWAS hits to causal genes will result in the same positive impact on drug approval. Despite this, genes prioritized by MendelVar may make for attractive targets for pharmaceutical companies as they may prove to be effective for more than one condition (i.e. both the common GWAS condition and the rarer Mendelian condition). Examples of successful drugs targeting Mendelian disease genes which are used to treat common conditions include PCSK9 antibodies and orexin antagonists. Monogenic forms of hypercholesterolaemia have implicated key genes in the LDL-cholesterol transport pathway, including *PCSK9*, with PCSK9 monoclonal antibodies evolocumab and alirocumab now indicated for treatment of common hyperlipidaemia. Dominant loss-of-function mutations in the orexin gene can cause narcolepsy; an orexin receptor antagonist drug (suvorexant) has proven effective for treating insomnia (Coleman *et al.*, 2017).

In this paper, we demonstrate the application of MendelVar to a selection of traits with different genetic architectures and ancestries of the population sample. Enrichment testing results highlight Mendelian disease genes with plausible links to tested traits. Based on the limited evidence of T1D versus T2D comparison, traits with lower-frequency and higher-effect size variants such as T1D may obtain better annotation from MendelVar, as previously shown (Freund *et al.*, 2018). Nevertheless, close to 1 in five loci in the T2D GWAS were assigned at least one candidate gene with relevant enriched disease symptoms, which remains highly informative. Standardized comparison of LDL and HDL GWAS across East Asian and European populations showed no consistent bias towards improved MendelVar annotation in either group. In fact, the majority of other trans-ancestry comparisons among adequately sized GWAS replicate common variant associations of origin predating human population divergence and tag the same loci with consistent direction and size of effect (Gurdasani *et al.*, 2019; Marigorta and Navarro, 2013; Shi et al., 2020). Overwhelming concordance has been confirmed between more recent blood lipid GWAS with high sample size in Europeans and trans-ancestry analysis of smaller sample size (Hu *et al.*, 2020).

The MendelVar approach to gene prioritization is limited by the number of genes with known Mendelian phenotypes (4843). As an example, we have examined the likely causal genes highlighted for T2D (Grotz *et al.*, 2017) and MendelVar identifies *PPARG*, *SLC30A8*, *ZMIZ1*, but not *KLF14*, which currently lacks a Mendelian disease assignment. The method used to define input intervals will also affect MendelVar’s findings. Our AD example using LD-defined intervals (*r*^2^>0.6) does not return the known causal gene *FLG* (Paternoster *et al.*, 2015) at its top locus but its paralog *FLG2* instead due to *FLG* placement outside the generated interval. Prior evidence shows that we might expect only about 83% of GWAS lead variants to be in tight LD (*r*^2^>0.8) with the causal gene, estimated (188/227) from known causal genes behind metabolite QTLs (Stacey et al., 2019), and so we have to expect that any method using LD to define a candidate gene list will not work for all loci. When defining the region purely by genomic distance, at 445 gold standard curated GWAS loci (Mountjoy et al., 2020) 90.9% of causal genes are within 100 kbp and 99.9% are within 1 Mbp, respectively, of the lead SNP and so MendelVar’s maximum interval size of ±10 Mbp will encompass the vast majority of causal genes.

As shown by examples of identification of candidate genes and enriched disease phenotypes/classes in T1D, T2D, LDL, HDL and atopic dermatitis GWAS, we envisage that MendelVar will be especially useful in the annotation and interpretation of GWAS results, but the flexibility of the user input enables other applications, such as in EWAS (epigenome-wide association studies). Ours and previous analyses (Freund *et al.*, 2018) underscore that MendelVar can be relied on to highlight enrichment of relevant biological processes implicated in investigated trait/disease. However, the degree of enrichment observed can vary depending on the relative contribution of Mendelian disease genes to a given trait.

MendelVar’s structured and comprehensive output allows inclusion into GWAS interpretation pipelines to complement other methods such as: Bayesian variant fine-mapping, variant pathogenicity prediction, molecular QTL mapping and colocalization, chromosome confirmation capture for identifying regulatory loops. As an example, we present *SERPINB7* as a new putative candidate gene in atopic dermatitis GWAS due to not only a phenotypic match between its target Mendelian disease and eczema but also due to colocalization evidence in the skin.

In summary, MendelVar provides a useful complementary method aiding candidate gene prioritization in GWAS of traits with various genetic architectures. MendelVar highlights disease processes, organ abnormalities and symptoms enriched among Mendelian disease genes which are likely dysregulated in phenotypically related complex traits.

## Funding

M.K.S., T.R.G. and L.P. work in a research unit funded by the UK Medical Research Council (MC_UU_00011/4). M.K.S. is funded by an Academy of Medical Sciences Springboard Award (awarded to L.P.), which is supported by the Wellcome Trust, The Government Department for Business, Energy and Industrial Strategy, the Global Challenges Research Fund and the British Heart Foundation [SBF003\1094].

*Conflict of Interest:* T.R.G. receives funding from GlaxoSmithKline and Biogen for unrelated research.

## Data availability

MendelVar webserver is publicly available on https://mendelvar.mrcieu.ac.uk.

Detailed MendelVar tutorial can be found on https://bitly.com/MendelVar or https://www.notion.so/mendelvar/MendelVar-tutorial-ab91d2a6acb846f2b9f2978fcd942dd5.

Code to reproduce MendelVar pipeline: https://github.com/MRCIEU/mendelvar_standalone.

Dataset S1. MendelVar annotation results for Onengut-Gumuscu et al. (2015) type 1 diabetes GWAS. https://doi.org/10.6084/m9.figshare.13154465.

Dataset S2. MendelVar annotation results for Xue *et al.* (2018) type 2 diabetes GWAS. https://doi.org/10.6084/m9.figshare.13154480.

Dataset S3. MendelVar annotation results for Teslovich *et al.* (2010) LDL GWAS. https://doi.org/10.6084/m9.figshare.13154567.

Dataset S4. MendelVar annotation results for Kanai *et al.* (2018) LDL GWAS. https://doi.org/10.6084/m9.figshare.13154525.

Dataset S5. MendelVar annotation results for Teslovich *et al.* (2010) HDL GWAS. https://doi.org/10.6084/m9.figshare.13154561.

Dataset S6. MendelVar annotation results for Kanai *et al.* (2018) HDL GWAS. https://doi.org/10.6084/m9.figshare.13154504.

Dataset S7. MendelVar annotation results for (sub)-significant loci in Paternoster *et al.* (2015) atopic dermatitis GWAS. https://doi.org/10.6084/m9.figshare.12174864.

NB. Datasets S1–S7 contain the following standard MendelVar output files:

- mendelvar.log: log file with MendelVar run parameters.
- input_file.bed: input submitted by user.
- disease_overlap.txt: overlap of user test intervals with Mendelian-disease causing genes.
- variant_overlap.txt: overlap of user test intervals with Mendelian-disease causing variants.

For each ontology tested for enrichment (DO, DO slim, HPO, HPO slim, Freund *et al.* (2018), GO, GO slim, Pathway Commons, Reactome, ConsensusPathDb), MendelVar provides:

*.out.inrich.parsed: file with processed INRICH results.
*.out.inrich: file with raw INRICH results.
*.pdf and *.png: summary enrichment figure.

## Supplementary Material

btaa1096_Supplementary_Data
