# Supplementary material for: MendelVar: gene prioritization at GWAS loci using phenotypic enrichment of Mendelian disease genes
Source: Bioinformatics. 2021 Jan 16;37(1):1–8. doi: 10.1093/bioinformatics/btaa1096 (PMC8034535; doi:10.1093/bioinformatics/btaa1096)
Supplement: btaa1096_Supplementary_Data [file btaa1096_supplementary_data.zip › MendelVar_supplementary_figs_tables.docx]

**Supplementary Figures and Tables**

**Figure S1.** Top enrichment results for Disease Ontology (DO) terms among Mendelian disease genes located within LD-based intervals around lead SNPs in Onengut-Gumuscu et al. (2015) type 1 diabetes (T1D) GWAS.

**
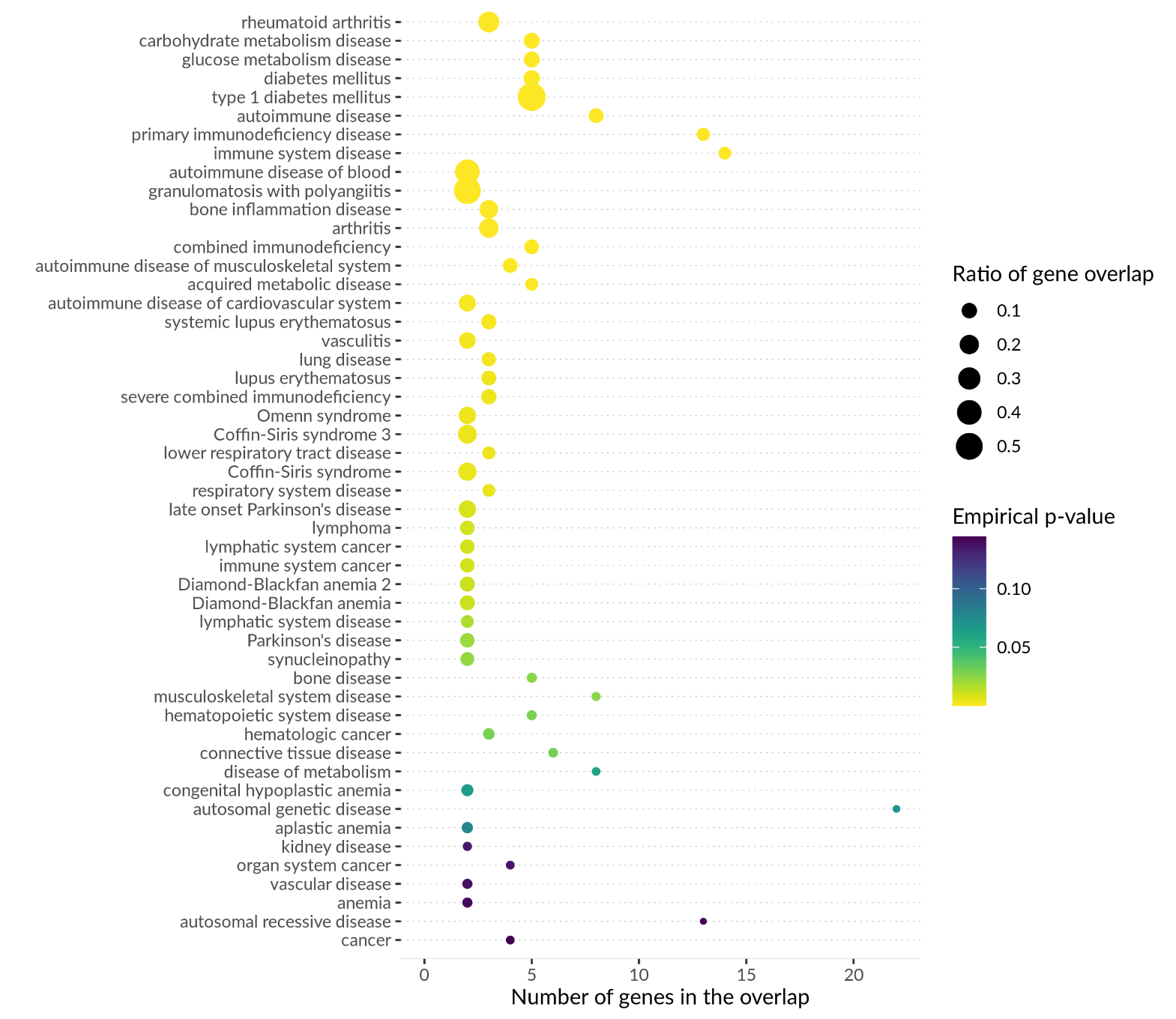
**

**
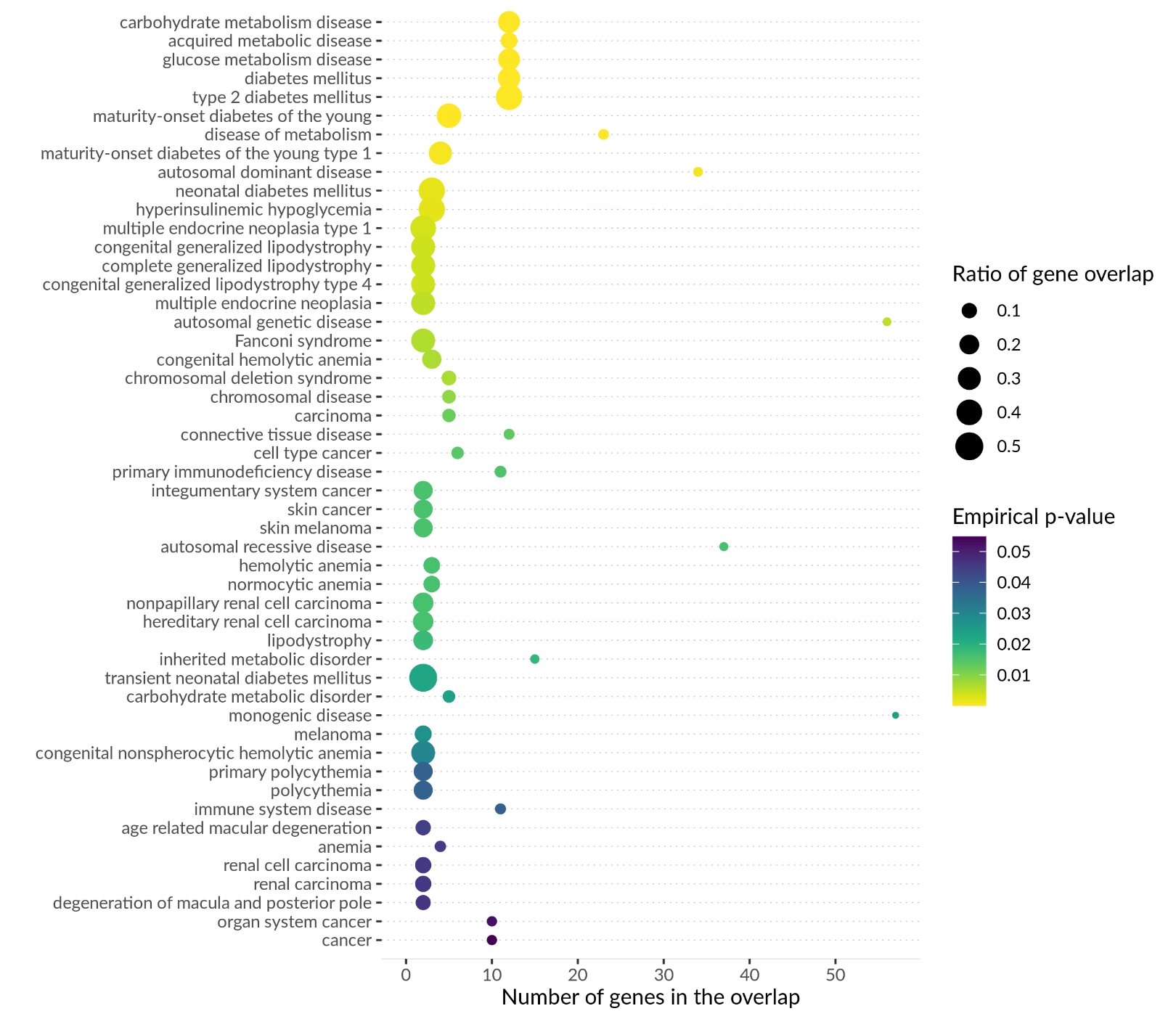
Figure S2.** Top enrichment results for Disease Ontology (DO) terms among Mendelian disease genes located within LD-based intervals around lead SNPs in Xue et al. (2018) type 2 diabetes (T2D) GWAS.

**Table S1.** Sample truncated output from the MendelVar disease gene overlap table. Each row (here transposed) in the table represents a single Mendelian disease gene overlapping our test interval.

|  | **Example** |
| --- | --- |
| **ID** | rs80293268 |
| **chrom** | chr1 |
| **interval_start** | 7647519 |
| **interval_end** | 8647519 |
| **gene_name** | CAMTA1 |
| **alias_symbol** | KIAA0833 |
| **hgnc_id** | 18806 |
| **Ensembl_gene_id** | ENSG00000171735 |
| **gene_start** | 6785454 |
| **gene_end** | 7769706 |
| **strand** | + |
| **omim_gene_id** | 611501 |
| **omim_disease_id** | 614756 |
| **orphanet_id** | 314647 |
| **disease_name** | Cerebellar ataxia, nonprogressive, with mental retardation |
| **hpo** | 0000752;0000718;0002120;0006919;0001263;0000276;0001260;0002317;0002019;0100540;0000307;0000490;0001348;0002403;0000414;0001319;0000160;0001321;0000639;0010867;0025517;0001249;0002003;0000445;0002470;0002536;0000369;0001256;0001310;0001250;0000729;0000494;0001251;0002080;0400005;0000179;0012433;0002275;0000343;0011067;0025191;0002354;0000750;0000337;0410170;0007256;0000463;0000316;0002020;0000256;0000411;0000486 |
| **do** | 50998 |
| **source** | Decipher;Genomics England;OMIM;Orphanet |
| **omim_link** | https://www.omim.org/entry/614756 |
| **disease_description** | Nonprogressive cerebellar ataxia with mental retardation is an autosomal dominant neurodevelopmental disorder characterized by mildly delayed psychomotor development, early onset of cerebellar ataxia, and intellectual disability later in childhood and adult life. Other features may include neonatal hypotonia, dysarthria, and dysmetria. Brain imaging in some patients shows cerebellar atrophy. Dysmorphic facial features are variable (summary by Thevenon et al., 2012). |

**Table S2.** Sample truncated output from the MendelVar variant overlap table. Each row (here transposed) in the table represents single ClinVar pathogenic/likely pathogenic/risk variant overlapping our test interval.

|  | **Example** |
| --- | --- |
| **ID** | rs80293268 |
| **chrom** | chr1 |
| **interval_start** | 7647519 |
| **interval_end** | 8647519 |
| **dbsnp_dbvar_id** | rs1135401818 |
| **variant_start** | 7677682 |
| **variant_end** | 7677682 |
| **cyto_location** | 1p36.23 |
| **ref_allele** | C |
| **alt_allele** | T |
| **effect** | Likely pathogenic |
| **HGSV_notation** | NM_015215.4(CAMTA1):c.2863C>T (p.Arg955Trp) |
| **gene** | CAMTA1 |
| **disease_name(s)** | Cerebellar ataxia, nonprogressive, with mental retardation |
| **disease_omim** | 614756 |
| **disease_orphanet** | NA |
| **clinvar_link** | http://www.ncbi.nlm.nih.gov/clinvar/variation/431151 |
| **VCV** | 431151 |
| **RCV** | 496160 |
| **allele_id** | 424639 |
| **quality_rating** | criteria provided, single submitter |

 **Table S3.** Comparison of MendelVar with related Mendelian disease- and enrichment- centred annotation tools.
