# Supplementary material for: MendelVar: gene prioritization at GWAS loci using phenotypic enrichment of Mendelian disease genes
Source: Bioinformatics. 2021 Jan 16;37(1):1–8. doi: 10.1093/bioinformatics/btaa1096 (PMC8034535; doi:10.1093/bioinformatics/btaa1096)
Supplement: btaa1096_Supplementary_Data [file btaa1096_supplementary_data.zip › MendelVar_supplementary_methods.docx]

**Online Supplementary Methods**

*Mendelian disease gene definition*

In MendelVar, we follow the working definitions of Mendelian disorders from Chakravorty & Hegde (2017) and Hansen et al. (2019) with broadly defined Mendelian genes to include genes causal for highly penetrant monogenic and oligogenic diseases, including diseases involving cell mosaicism, recurrent de novo structural variants causing mostly developmental diseases, such as Smith-Magenis syndrome and some germline cancer susceptibility genes such as BRCA1/BRCA2.

As long as the gene-phenotype association is highly replicable, penetrant, rare, and of large effect, it increases our understanding of biological processes involved in disease pathology and augments our pool for potential therapeutic targets. As such it is included in MendelVar, in line with inclusion in the main reference for knowledge about Mendelian disease – the OMIM database. Still, the majority of relationships presented stick to the strict monogenic Mendelian model (5,686 traits on OMIM, October 2020), followed by susceptibilities to complex disease or infection (693 traits) and somatic cell genetic disease (230 traits).

*Gene annotations*

APPRIS (Rodriguez et al., 2013) isoform definitions were downloaded from its website along with the matching GENCODE GFF annotation files: v19 (for GChr37) and v31 (for GChr38). We used the canonical APPRIS transcripts to define gene coordinates. When more than one canonical isoform was available in APPRIS, we chose the longest transcript. Only 20,738 coding genes are present in APPRIS. Around 37,000 more non-coding genes are annotated in GENCODE - RNA genes, hypothetical genes etc. which do not have a defined CDS. We included those genes in our dataset by selecting the longest transcript available in GENCODE.

*Database input filtering*

OMIM filtering was as follows: disease gene mapping key must be 3 (also prefix #), ie. with known molecular basis - known causal gene. Gene-disease relationships reported in DECIPHER DDG2P were kept if they were in category: “probable” or “confirmed” but not “possible”.

Data from DECIPHER and Orphanet required additional cleaning, in terms of checking presence of the disease/gene MIMs in the OMIM database and their up-to-date status – some MIM IDs needed upgrading from deprecated to current IDs. We also included checks to establish congruence between MIM gene IDs and gene symbol and complete missing MIM gene IDs.

*Integration of OMIM, Orphanet, DECIPHER and Genomics England PanelApp*

The four different data sources for Mendelian disease-gene mapping (OMIM, Orphanet, DECIPHER, Genomics Panel App) were subsequently integrated. Genes were all standardised by HGNC ID (Eyre et al., 2006), Ensembl IDs, and HGNC approved symbol (in that order), and disease names through OMIM IDs, Orphanet IDs and OMIM names.

*ClinVar variant data processing*

Variants downloaded from ClinVar FTP in the variant_summary.txt.gz file were filtered to retain only those that contain "pathogenic", "likely pathogenic" or "risk factor" among their effects. Variants spanning many genes were eliminated, to keep variants directly linked to a single or a very small number of genes. Variants missing coordinates were also discarded, and phenotype descriptions matched to MIM IDs whenever possible. This filtering strategy resulted in retention of approximately 20% of variants – 105,824 and 104,099 in GRCh37 and GRCh38, respectively.

*Ontology processing*

For multi-level ontologies – DO, HPO, GO, we propagated all the transitive “is_a”, “part_of” relationships up to the root using the R package ontologyIndex (Greene, Richardson, & Turro, 2016), and included all the parent terms in addition to leaf terms, exclusive of the root terms in each ontology, because these are uninformative.

We eliminated all Inferred from Electronic Annotations (IEA) and entries with “NOT” qualifier (ie. gene is NOT characterised by the term), as IEA annotations are not manually curated, often inferred just by text mining algorithms and can be thus unreliable.

We only retained the “Phenotypic abnormality” HPO ontology and discarded children of HP:0000005 Mode of inheritance, HP:0031797 Clinical course, HP:0040279 Frequency, HP:0012823 Clinical modifier as these encompass a small number of child terms and do not correlate with the mechanistic basis of disease. HPO slim ontology was created by pruning the tree to only 25 direct descendants of the root HP:0000118 Phenotypic abnormality term.

In REACTOME, we used the Ensembl2Reactome_All_Levels.txt file which contains gene annotations across all pathway levels.

Finally, we subsetted all the ontologies only to the Mendelian disease genes in the MendelVar database and eliminated ontology annotations of the genes with no evidence for disease causality, as we want to test for term enrichment relative to genes linked to Mendelian disease rather any gene as background.

*Enrichment testing with INRICH*

INRICH was revealed to be one of the most sensitive and specific methods for conducting overrepresentation analysis on GWAS data (De Leeuw, Neale, Heskes, & Posthuma, 2016) and is a fast C++ software, which makes it ideal for our application.

INRICH takes a list of associated genomic intervals and tests for enrichment against gene sets. The intervals need not be independent from each other, as overlapping intervals are merged, as well as overlapping genes belonging to the same gene set (Lee et al., 2012). Background interval sets for enrichment testing are permuted to match the input set in terms of the number of SNPs, SNP density and number of overlapping genes which removes a lot of bias when using GWAS-derived genomic intervals.

**Resources**

Gencode:

*GRCh38/hg38*

<ftp://ftp.ebi.ac.uk/pub/databases/gencode/Gencode_human/release_31/gencode.v31.annotation.gff3.gz>

*GRCh37/hg19*

<ftp://ftp.ebi.ac.uk/pub/databases/gencode/Gencode_human/release_19/gencode.v19.annotation.gff3.gz>

HUGO Gene Nomenclature Committee (HGNC)

<ftp://ftp.ebi.ac.uk/pub/databases/genenames/new/tsv/hgnc_complete_set.txt>

APPRIS isoforms

*For version hg19 (matched to Gencode version 19)*

<http://apprisws.bioinfo.cnio.es/pub/current_release/datafiles/homo_sapiens/GRCh37/appris_data.principal.txt>

*For version hg38 (matched to Gencode version 31)*

<http://apprisws.bioinfo.cnio.es/pub/current_release/datafiles/homo_sapiens/GRCh38/appris_data.principal.txt>

1000 Genomes

*GRCh37/hg19*

<http://ftp.1000genomes.ebi.ac.uk/vol1/ftp/release/20130502/>

*GRCh38/hg38*

<http://ftp.1000genomes.ebi.ac.uk/vol1/ftp/release/20130502/supporting/GRCh38_positions>

HapMap II recombination hotspots

<ftp://ftp.ncbi.nlm.nih.gov/hapmap/recombination/2011-01_phaseII_B37/genetic_map_HapMapII_GRCh37.tar.gz>

LDlink LDproxy

<https://ldlink.nci.nih.gov/?tab=ldproxy>

UCSC liftOver

<https://genome.ucsc.edu/cgi-bin/hgLiftOver>

Giggle

<https://github.com/ryanlayer/giggle>

INRICH

<https://atgu.mgh.harvard.edu/inrich/>

OMIM

<https://www.omim.org/api>

Orphanet

<https://www.orpha.net/>

*Rare diseases and cross referencing*

<http://www.orphadata.org/data/xml/en_product1.xml>

*Linearisation of disorders*

<http://www.orphadata.org/data/xml/en_product7.xml>

*Rare diseases with their associated genes*

<http://www.orphadata.org/data/xml/en_product6.xml>

Genomics England

<https://panelapp.genomicsengland.co.uk/api/v1/>

DECIPHER

<https://decipher.sanger.ac.uk/>

<http://www.ebi.ac.uk/gene2phenotype/downloads/DDG2P.csv.gz>

<https://www.ebi.ac.uk/gene2phenotype/downloads/EyeG2P.csv.gz>

<https://www.ebi.ac.uk/gene2phenotype/downloads/SkinG2P.csv.gz>

ClinVar

<https://www.ncbi.nlm.nih.gov/clinvar/>

<ftp://ftp.ncbi.nlm.nih.gov/pub/clinvar/tab_delimited/variant_summary.txt.gz>

Disease Ontology

<http://disease-ontology.org/>

*Full ontology*

<https://raw.githubusercontent.com/DiseaseOntology/HumanDiseaseOntology/master/src/ontology/doid-merged.obo>

*Slim ontology*

<https://raw.githubusercontent.com/DiseaseOntology/HumanDiseaseOntology/master/src/ontology/subsets/DO_AGR_slim.obo>

Human Phenotype Ontology

<https://hpo.jax.org/>

*Ontology*

<https://raw.githubusercontent.com/obophenotype/human-phenotype-ontology/master/hp.obo>

*Annotation*

<http://compbio.charite.de/jenkins/job/hpo.annotations.current/lastSuccessfulBuild/artifact/current/phenotype.hpoa>

Freund et al. gene sets

<https://github.com/bogdanlab/gene_sets/tree/master/mendelian_gene_sets>

Gene Ontology

*Full ontology*

<http://current.geneontology.org/ontology/go-basic.obo>

*Slim ontology*

<http://current.geneontology.org/ontology/subsets/goslim_generic.obo>

*Annotation*

<ftp://ftp.ebi.ac.uk/pub/databases/GO/goa/HUMAN/goa_human.gaf.gz>

ConsensusPathDB

<http://consensuspathdb.org/>

Pathway Commons

<https://www.pathwaycommons.org/>

<https://www.pathwaycommons.org/archives/PC2/v12/PathwayCommons12.All.hgnc.gmt.gz>

Reactome

<https://reactome.org/>

<https://reactome.org/download/current/Ensembl2Reactome_All_Levels.txt>

**Supplementary References**

Chakravorty, S., & Hegde, M. (2017) Gene and variant annotation for Mendelian disorders in

the era of advanced sequencing technologies. *Annu. Rev. Genom. Hum. G.,* *18*(1), 229–256.

De Leeuw,C.A. *et al.* (2016) The statistical properties of gene-set analysis. *Nat. Rev. Genet.*, *17*(6), 353–364.

Eyre,T.A. *et al.* (2006) The HUGO Gene Nomenclature Database, 2006 updates. *Nucleic Acids Res.*, *34*(S1), D319–D321.

Greene,D. *et al.* (2016) ontologyX: a suite of R packages for working with ontological data. *Bioinformatics*, *33*(7), 1104–1106.

Hansen, A. W. *et al.* (2019) A Genocentric approach to discovery of Mendelian disorders. *Am. J. Hum. Genet*, *105*(5), 974-986.

Lee,P.H. *et al.* (2012) INRICH: Interval-based enrichment analysis for genome-wide association studies. *Bioinformatics*, *28*(13), 1797–1799.

Rodriguez,J.M. *et al.* (2013) APPRIS: Annotation of principal and alternative splice isoforms. *Nucleic Acids Res.*, *41*(D1), 110–117.
